# Narrowing down the distal border of the copy number variable beta-defensin gene cluster on human 8p23

**DOI:** 10.1186/1756-0500-7-93

**Published:** 2014-02-19

**Authors:** Stefan Taudien, Klaus Huse, Marco Groth, Matthias Platzer

**Affiliations:** 1 Leibniz Institute for Age Research – Fritz Lipmann Institute, Beutenbergstr. 11, D-07745 Jena, Germany

**Keywords:** Defensin, CNV borders, Length polymorphism, Paralog ratio test, Indel, *DEFB108P*, *DEFB109P*

## Abstract

**Background:**

Copy number variation (CNV) in the range from 2 to 12 per diploid genome is an outstanding feature of the beta-defensin gene (DEFB) cluster on human chromosome 8p23.1 numerously demonstrated by different methods. So far, CNV was proven for a 115 kb region between *DEFB4* and 21 kb proximal of *DEFB107* but the borders for the entire CNV repeat unit are still unknown. Our study aimed to narrow down the distal border of the DEFB cluster.

**Results:**

We established tests for length polymorphisms based on amplification and capillary electrophoresis with laser-induced fluorescence (CE-LIF) analysis of seven insertion/deletion (indel) containing regions spread over the entire cluster. The tests were carried out with 25 genomic DNAs with different previously determined cluster copy numbers. CNV was demonstrated for six indels between ~1 kb distal of *DEFB108P* and 10 kb proximal of *DEFB107.* In contrast, the most distal indel is not affected by CNV.

**Conclusion:**

Our analysis fixes the minimal length of proven CNV to 157 kb including *DEFB108P* but excluding *DEFB109P.* The distal border between CNV and non-CNV part of the DEF cluster is located in the 59 kb interval chr8:7,171,082-7,230,128.

## Background

**Figure 1:**
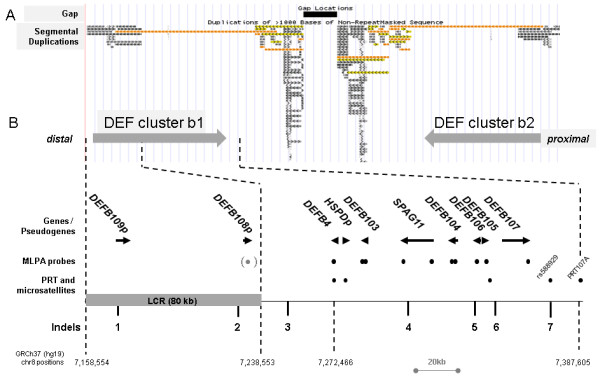
**Beta-defensin cluster on human chromosome 8p23.1. (A)** In the UCSC Genome Browser (GRCh37 hg19 chr8:7,158,554-7,855,043), two gene clusters are arranged on both sides of a clone gap and in opposite direction, illustrated by the grey arrows and named DEF cluster b1 and b2. Segmental duplications are flanking the clusters or are part of it. **(B)** Zoom of DEF cluster b1 with genes / pseudogenes and the distally located 80 kb low copy repeat (LCR) previously described [9]. Dots indicate MLPA probes, PRT amplicons and microsatellites used for individual DEF cluster b CN determination by us and others [5,26,33]. The MLPA probe for DEFB108P (grey, in parantheses) was removed from the current kit version due to inconsistent results. Indels investigated in this study to narrow down the CNV borders are numbered from 1 to 7.

The beta-defensin gene cluster (DEF cluster b) on human chromosome 8p23.1 is one of the most prominent examples for functional-relevant copy number variation (CNV) and has been intensively investigated over the last years. Ten genes and pseudogenes (*DEFB109P, DEFB108P, DEFB4, HSPD1P, DEFB103, SPAG11, DEFB104, DEFB106, DEFB105* and *DEFB107*) are organized in a cluster of ~218 kb embedded in different types of low copy repeats (LCR) originating from segmental duplications. Furthermore, the DEF cluster b is embedded in one of two complex segmental duplications, REPD and REPP, involved in polymorphic inversions [1-3]. Recently, in these regions also CNV for the FAM90A gene class was shown [4]. Due to this repetitive structure which is refractory to analysis, the locus is one of the few regions with a remaining recalcitrant gap even in the most recent human genome assembly GRCh37 (hg19, February 2009). In this assembly, two clusters - DEF cluster b1 and b2 (hg19 chr8:7,170,368-7,366,833 and chr8: 7,669,242-7,855,043, respectively) - are arranged in opposite direction on both sides of the gap and slightly differ in consensus sequences (Figure 1A). In the following, for convenience, genomic positions are given only for the distal defensin gene cluster b1. Since the assembly is based on clones from different individuals, the arrangement is arbitrarily, in fact there is variation of the cluster copy number (CN) between 2 and 12 among individuals which is accompanied by sequence differences between clusters and individuals [5-11]. Hence, determination of individual DEF cluster b CNs is of basic interest, namely due to putative or proven association or non-association of the CN with multiple diseases [12-24]. Even after years, CN estimation is still a technical and methodological challenge [25]. From different technologies, Multiplex Ligation-dependant Probe Amplification (MLPA) and Paralog Ratio Tests (PRT) have emerged as the most accurate ones [26-30].

By MLPA, Groth et al. have proven concordance of the CN for all genes which were represented by probes [5]. The kit used at the time (P139, MRC Holland http://www.mrc-holland.com) targeted 8 genes of the cluster. For 7 genes (*DEFB4, DEFB103, SPAG11, DEFB104, DEFB106, DEFB105* and *DEFB107*), the probes hybridize to regions with no other paralogs than the DEF cluster b copies on 8p23.1 (Figure 1B). In contrast, the eighth target *DEFB108P* is located in a previously identified 80-kb low copy repeat, named LCR IV [9], with additional paralogs on chromosome 4, 11 and 8 (~12.0 and 12.2 Mb). Since there is no evidence for transcription except for an imperfectly mapping partial coding sequence (AF540980), the paralogs in the DEF cluster b are most probably pseudogenes, whereas the chromosome 11 paralog is the *DEFB108* gene according to the HUGO nomenclature committee (HGNC29966). The MLPA results for *DEFB108P* were not reproducible and the probe was removed from the kit when changing to kit version P139-B1 in June 2009.

The basic idea of PRT for CN estimation is to co-amplify with a single primer pair CNV regions together with paralogous loci not affected by CNV [31]. Presuming equal amplification efficiency for all paralogs due to the common primers, the non CNV affected ones serve as calibrators assuming two copies per paralog and diploid genome (in case of a single calibrator locus) and allow the calculation of CN for the variable paralogs. CNV- and non-CNV paralogs can be distinguished either by sequence differences or - in case of indels - by length differences of the PCR products.

DEF cluster b as the region of interest is characterized by two opposite features with regard to the existence of paralogous regions (Figure 1). The LCR containing *DEFB109P* and *DEFB108P* has six paralogs at non-CNV loci on different chromosomes [9]. For sub-regions in the size range of PRT suited PCR products (~200-500 bp) even 12 paralogs exist with nucleotide identities between 85 and 99%. This makes the establishment of a reliable PRT difficult since equal amplification of all paralogs is questionable. To circumvent the problem, the number of co-amplified non-CNV paralogs can be reduced to one or two by skillful primer design.

In contrast, in the interval from the proximal LCR end to the clone gap, only few short regions with paralogs outside the DEF cluster exist which can be used as calibrators in PRTs for CN determination. CN estimations by PRTs applied by us and others [5,6,20,27] as well as the triplex-PRT by Aldhous et al. [26] are targeting CN-invariant pseudogenes of the heat shock 60 kDa protein (*HSPD1*; NCBI accession no. NM_002156) on several chromosomes (GENCODE pseudogene IDs: PGOHUM00000X with X: 239058, 246095, 235528, 237584, 246095, 235528, 239809 [32]) and the CNV paralog between *DEFB4* and *DEFB103* (*HSPD1P3*; PGOHUM00000249546; hg19 chr8: 7,276,513-7,278,068). Furthermore, Abu Bakar et al. applied a combination of analyses including PRT, microsatellites and a multiallelic length polymorphism to determine individual CN with PRT107A as the most proximal interrogated locus, located 21 kb proximal of *DEFB107*[33].

Initially, the minimal length of the CNV region was estimated to ~240 kb by pulsed-field gel electrophoresis of *Sfi*I-digested genomic DNA after hybridization with *SPAG11* and *DEFB4* probes [8] but, in a strict sense, CNV is proven for 115 kb between the most distal MLPA probe in *DEFB4* and the PRT107A amplicon (hg19 chr8: 7,272,466 - 7,387,605). While the first assay is rather a conservative valuation, the latter is assumed to underestimate the CNV length. Respectively, it is currently not known whether the pseudogenes *DEFB108P* and *DEFB109P* are included in the CNV region or not.

With respect to these facts, the aim of the present study was to narrow down the borders of CNV region of the DEF cluster b. Their more precise determination should allow conclusions about the CNV mechanism, the degree of variation between individual DEF cluster b repeat units and help to elucidate the assumed impact of the direction of the cluster copies. To this end, either single nucleaotide variations (SNV) or insertions/deletions (indels) are suitable. Allele ratios for SNVs, however, are much harder to quantify, e.g. by pyrosequencing, than it can be done for indels by length differences in electrophoresis where quantification is done on separated size-defined single strands (CE-LIF). Accuracy and reproducibility is lower as proven by us and others analyzing samples with verified DEF cluster copy numbers. For example, CN estimation of 41 samples by a pyrosequencing based paralog ratio test (PPRT) resulted in 21 CN underestimations of up to 2 copies (Table two in [5]). Another PPRT resulted in better results, but also revealed 3 underestimations out of 14 analyses (Table one in [34]). Therefore, we established a set of length polymorphism tests based on PCR of regions with small indels. Application of these tests to genomic DNAs from individuals with previously determined DEF cluster CNs and estimation of the peak area ratios provide information whether the analyzed indels are affected by CNV or not.

## Results

In order to confirm or exclude CNV at multiple sites spread over DEF cluster b, namely distal and proximal of the outermost ones with proven CNV, we identified indels in the DEF cluster b by two approaches. We first checked the SNP database (NCBI dbSNP Build 137; June 26, 2012) in the DEF cluster b1 (hg19 chr8:7,170,368-7,366,833) for indels of at least 3 nucleotides and located outside of homonucleotide stretches. Furthermore we retrieved all indels with these features previously identified by 454/Roche sequencing of four DEF cluster enriched genomes with known CN ([11] and unpublished results). This resulted in 50 indels, of which 23 were located in repetitive regions complicating primer design. Despite this we selected 23 loci (11 repetitive, 12 non-repetitive) for further analysis that cover as evenly as possible regions of DEF cluster b not yet tractable by MLPA or established PRTs. Consistent results were obtained for seven indels (2 repetitive, 5 non-repetitive), named indel 1 to 7 in the order from the distal to the proximal end of DEF cluster b1 (Figure 1B, Additional file 1: Table S2 and S3). Three-fold replication of the assays with 96 DNAs resulted in unambiguous accordance of the allele ratios with the MLPA determined CNs and standard deviations for the area ratios <0.15 (data not shown). The CE-LIF results for the remaining loci were not reproducible, showed fragments of unexpected lengths and/or the calculated fragment ratios were incompatible with the known CN of the test samples (data not shown).

Indels 3, 6 and 7 are located in intergenic regions, indels 1 and 5 in intron 1 of *DEFB109P* and *DEFB106*, respectively, and indel 2 is 273 bp upstream of the *DEFB108P* start codon. Five indels are known variations from dbSNP, indels 1 and 2 are novel ones and were submitted to dbSNP. The amplicon lengths range between 255 and 396 bp, the length differences between indel variants count for 3 to 5 nucleotides.

### Known indels 3 to 7

Indels 3 to 7 are located in regions for which no other paralogs exist than the DEF cluster copies themselves. Hence, useful for the proof of CNV are only samples with CN > 2 that harbor clusters with both indel variants in unequal numbers. Analyses of these samples result in two CE-LIF signals with peak area ratios unequal 1:1. For example, a peak area ratio (short:long) of 1:2 = 0.5 is produced by one DEF cluster copy with the short and two copies with the long variant (or a multiple thereof). Consistence with previously determined MLPA CNs 3 or 6 per diploid genome would confirm a concordant CN at the indel site. For all indels 3 to 7, between 5 (20%) and 9 (36%) of the 25 samples per indel were informative in this way (Additional file 1: Table S4). Though this is a modest rate it has to be noted that the assays are neither thought nor suitable to serve as a CN estimation method but rather to find the borders of the CN variable genomic region. In total, 34 of 125 CE-LIF electropherograms (27%) showed peak area ratios unequal 1:1 and consistent with the diploid CN previously determined by MLPA (Table 1). This evidences CNV for the positions of indels 3 to 7.

**Table 1:** Peak area ratios unequal 1:1 obtained by CE-LIF assays of indels 3 to 7 using 25 genomic DNAs with known diploid copy numbers (CN)

**Diploid CN by MLPA**	**Peak area ratios short/long**	**Cluster ratio short/long**	**Indel no**	**# of informative tests**
3	1.92-2.07	2:1	3,5,6	6
	0.50-0.54	1:2	3,4,5,6	6
4		1:3		---
		3:1		---
5	0.29	1:4	7	1
	0.68/0.72	2:3	4,5	2
	1.48	3:2	7	1
		4:1		---
6		1:5		---
	0.47-0.58	2:4	4,5,6,7	7
	2.00/2.12	4:2	3	2
		5:1		---
7	0.16	1:6	7	1
	0.43	2:5	6	1
	0.60/0.76	3:4	5,7	2
		4:3		---
		5:2		---
		6:1		---
8		1:7		---
	0.41/0.46	2:6 or 3:5	5,7	2
	0.77	3:5	5	1
	1.53	5:3	7	1
	2.75	6:2	7	1
		7:1		---
Total No of tests, informative for proof of CNV				34

Between five and nine DNAs per indel showed peak area ratios unequal 1:1 (Additional file 1: Table S3), resulting in a total of 34 electropherograms proving CNV. Indels 4 to 6 are located between *DEFB4* and *DEFB107* and therefore confirm CNV already proven by MLPA. Indels 3 and 7 demonstrate CNV outside this region (see Figure 1).

### Novel indels 1 and 2

In contrast to indels 3 to 7, indels 1 and 2 are located in the LCR and PCR also amplifies paralogs from loci not affected by CNV on chromosomes 4, 8, 11 and 12. This resembles the situation of established PRTs but requires a strict discrimination of CN variable and invariable regions by the indel variants. To check this as comprehensive as possible, we retrieved the sequences of all indel 1 or 2 containing BAC clones from GenBank and monitored the variant status (short or long) of the indel sites. We identified 10 and 22 clones containing indels 1 and 2, respectively, derived from five BAC libraries (RPCI-11, RPCI-13, CTD, CHORI-17, SCb, Additional file 1: Table S5). In addition we mapped the clone sequences against the human genome assembly GRCh37 (hg19) by Blast and registered the chromosomal location with the highest score. For comparison we also checked the chromosomal assignments of the clones in the nucleotide database entry, in the UCSC genome browser and in a locus specific assembly [9]. For indel 1 we found six clones with the short variant, doubtlessly assigned to DEF cluster b1 or b2, and three clones with the long variant mapping to chromosome 12. However, we also identified one BAC (AF252830) with the long variant which is not represented in the GRCh37 (hg19) assembly but maps to DEF cluster b according to the Blast result and the previous assignment [9]. From the clones harboring indel 2, 11 were identified with the short variant, unambiguously mapping to the DEF cluster. 9 clones have the long variant and map to the paralogous regions on chromosomes 4, 8 (12.0 and 12.3 Mb) or 11. In contradiction to the presumption, two clones (AC130379.8 and AC092111.9) with the short variant were found mapping to chromosome 8 (12.3 Mb) and chromosome 12, respectively. These data indicate that neither indel 1 nor indel 2 are suited to establish a quantitative PRT. Nevertheless, the striking overrepresentation of the short variants of both indels among DEF cluster copies prompted us to establish CE-LIF assays for the qualitative assessment of CN variability.

For indel 1, paralogs are located on chromosomes 4 (9.19 Mb), 8 (12.01 and 12.26 Mb) and 12 (8.36 Mb). To reduce the number of co-amplified paralogs we used a reverse primer with complete match to the DEF cluster and the chromosome 12 paralog but 3’ mismatches to the paralogs on chromosome 4 and 8 (12.01 and 12.26 Mb) (Additional file 1: Figure S6). The amplified region for indel 2 has paralogs on chromosomes 4, 8 11 and 12. In an analogous manner as for indel 1 we aimed to avoid the amplification of the paralogs on chr8 (7.17 / 7.86 / 12.02 / 12.26 Mb) and chr12 (8.36 Mb) by designing a forward primer matching at his 3’end only those on chromosome 4 (9.40 Mb), 8 (11.96 / 12.20 Mb), 11 (71.54 Mb) and the DEF cluster (Additional file 1: Figure S7). In order to check how successful the designed primers are in reducing the number of amplified paralogs, we cloned the PCR products of indel 1 (three samples) and indel 2 (two samples), sequenced between 24 and 43 clones per indel and sample and assigned the sequences to chromosomal positions according to GRCh37 (hg19) (Additional file 1: Table S8). For indel 1 we obtained exclusively clones from the expected paralogs (DEF cluster b and chromosome 12). None of the clones were assigned to the undesired paralogs on chromosome 4 and 8 (12.01 and 12.26 Mb) but 6 out of 85 sequences (~7%) represented chimera derived from the DEF cluster b and chromosome 12. Similarly, sequencing clones from indel 2 revealed only clones derived from the expected paralogs of the DEF cluster and paralogs on chromosomes 4, 8 (11.95 and 12.20 Mb) and 11. Furthermore, we found 3 out of 80 clones (~4%) to be chimeric derived from chromosome 4 and 11. For both indel 1 and 2 we observed in all non-chimeric clones exclusively the short variant for the DEF cluster but the long one for the paralogs, confirming the unequal distribution of the indel variants among BACs derived from CNV and non-CNV loci.

Based on these results we concluded that the short/long ratio determined from the CE-LIF electropherograms for samples with different DEF cluster CNs should be indicative for CN variability. A positive correlation of the variant ratio with the CN would confirm CNV whereas a rather constant ratio would indicate CN invariance.

**Figure 2:**
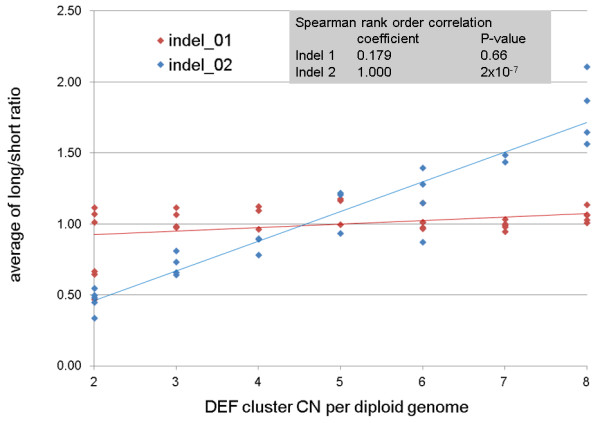
Long/short ratios for indel 1 (red) and indel 2 (blue) for the CN classes 2 to 5.

In agreement with this assumption, the results of the analyses for both indels (Figure 2, Additional file 1: Table S9) suggests that indel 2 (Spearman rank order correlation coefficient 1.00; P = 2x10^-7^) but not indel 1 (0.179 / 0.66) is included in the CNV region of the DEF cluster.

## Discussion

By PCR amplification and CE-LIF analysis of seven loci with small indels spread over DEF cluster b we could show that:

•CNV is confirmed by indels 4 to 7 for the region between *DEFB4* and 10 kb proximal of *DEFB107* which is in agreement with MLPA and PRT results;

•beyond this region CNV is proven by indels 2 and 3 in distal direction until hg19 chr8:7,230,128 thereby including the *DEFB108P* pseudogene in the CNV region of the cluster;

•*DEFB109P* is not comprised in the CNV region as proven by the indel 1 analyses.

These results suggest that the distal border of CN alleles with more than one DEF cluster is located in the interval of 59,047 bp between the amplicons for indel 1 and 2, i.e. in the region hg19 chr8:7,171,082-7,230,128 (DEF cluster b1) and chr8:7,796,830-7,854,329 (DEF cluster b2).

We tried to further narrow down the exact border of CNV in this region by analysis of three additional indels located between hg19 chr8:7,185,414 and chr8:7,200,941 but obtained inconsistent results most likely due to the co-amplification of more than the expected paralogs (data not shown).

The proof of CNV for indel 7, the most proximal variation analyzed by us, is in agreement with previously published experiments by Abu Bakar et al. using the multiallelic length polymorphism rs5889219 for CN determination [33]. This analysis targets a region overlapping with our indel 7 assay. Furthermore, the authors confirmed CNV by a PRT amplifying hg19 chr8:7,387,449-7,387,605 (named PRT107A) which is ~11 kb proximal of indel 7. We attempted to identify indels further from this position in proximal direction, i.e. until the clone gap at hg19 chr8:7,474,650. In the SNP database (dbSNP build 137) we found 23 indels according to our criteria (>2 bp length difference of the variants and not located in homonucleotide stretch). However, we were unable to develop suitable length polymorphism tests due to the repetitive sequence structure around these indels. The designed primer pairs resulted in *in-silico*-PCR products derived from up to 35 paralogs of >90% nucleotide identity.

Therefore, we conservatively estimate the minimal length of the CNV region to 157,478 bp (indel 2 – PRT107A).

Our approach in the LCR region of DEF cluster b is compromised by the extremely repetitive structure resulting in the co-amplification of numerous paralogs from all over the genome. Moreover, formation of chimeric products must always be taken into account when performing PCR in regions with high nucleotide identities.

By skillful primer design we tried to restrict the number of amplified paralogs to a minimum, ideally to one. As shown by cloning and sequencing of selected PCR products, for indel 1 only one paralog on chromosome 12 was amplified together with the DEF cluster b copies on chromosome 8. For indel 2, we could reduce the number of co-amplified paralogs from nine to four. Furthermore, we found chimeric sequences in the PCR products of both indels. These processes, however, may have contributed to examples with high deviation of the measured peak area ratios for samples of the same CN (Figure 2).

Moreover, the analysis of BAC sequences showed that the two indel alleles are not exclusively linked to either the CNV or the non-CNV LCRs. Thus, individual genomes may contain deviating indel alleles both in the DEF cluster b copies as well as in the paralogs used as calibrators. The presence of such “deviating copies” in one ore more of the analyzed samples might also have contributed to the observed high deviations.

Due to all these limitations, the test for indel 2 is not suitable for a reliable CN estimation of DEF cluster b but the strong correlation between the long/short variant’s ratio and CN unambiguously proves inclusion of indel 2 in the CN variable part of the region. Since it is located 273 bp distal of *DEFB108P*, CNV is also proven for the pseudogene.

Because we demonstrated the lack of CNV for indel 1 and the *DEFB109P* pseudogene, the distal border that separates the unique, CN invariable from the CN variable part of the DEF cluster must be located in the interval between both indels.

## Conclusion

Our analysis fixes the minimal length of proven CNV to 157 kb including *DEFB108P* but excluding *DEFB109P.* The distal border between CNV and non-CNV part of the DEF cluster is located in the 59 kb interval chr8:7,171,082-7,230,128. A more exact position remains to be identified as well as the site of the proximal border does. Due to the repetitive character of these borders it may also be possible, that borders vary between cluster copies and CN alleles which would further complicate a final evaluation. In any case, our findings underscore the mechanistic role of the LCRs bordering the DEF cluster in origin and maintenance of that exceptional CNV locus.

## Methods

### DNAs

DNAs are originated from: (1) The CARLA study [35,36], a prospective cohort study of the general elderly population (21 samples); (2) Healthy blood donors [37] (2 samples); (3) Coriell cell lines (2 samples). DNA-preparation and DEF cluster b CN estimation by MLPA was performed as previously described [5,23]. CNs are listed in Additional file 1: Table S1.

### PCR

~100 ng DNA were amplified in a total volume of 25 uL using the BioMix (Bio-25012) PCR-Premix (Bioline GmbH Luckenwalde, Germany) with 1 pmol of each primer listed in Additional file 1: Table S2, supplied by Metabion (Martinsried, Germany). The reverse primers were 5’-6-carboxyfluorescein (FAM)-labeled. The PCR conditions were as follows: pre-denaturation at 95°C for 2 minutes, 30 cycles of denaturation at 95°C for 30 seconds followed by annealing at 52°C for 30 seconds and elongation at 72°C for 1:30 minutes, final elongation at 72°C for 30 minutes and cooling at 18°C for 2 minutes.

### Capillary electrophoresis with laser-induced fluorescence (CE-LIF)

The FAM-labeled PCR products were appropriately diluted (between 1/30 and 1/500) and 1 μl dilution was supplemented with 10 μl HiDi-formamide (Roth, Karlsruhe, Germany) and 0.5 μl of GeneScan ROX 500 size standard (Applied Biosystems, Darmstadt, Germany). The mixture was denatured at 94°C for 3 min, and subsequently cooled on ice. The denatured products were then separated on an ABI 3730 capillary sequencer and analyzed with the GeneMapper 4.0 software (Applied Biosystems, Darmstadt, Germany).

### Cloning and Sanger sequencing (indels 1 and 2)

PCRs were carried out as described above with unlabeled primers using the DNAs 1105126, 3208698, 2121707 (indel 1) and 1101938, 3216315 (indel 2). The PCR products were cloned into pCR2.1 by the TOPO TA Cloning Kit (Invitrogen, Darmstadt, Germany) according to the manufacturer's instructions. Colonies were picked (indel 1: 32 for each DNA / indel 2: 48 for each DNA) and grown in LB Broth supplemented with ampicillin. Plasmid DNA was isolated from the cultures by automated Qiaprep (Qiagen, Hilden, Germany) and inserts were sequenced on a capillary sequencer ABI 3730 using the M13 reverse universal primer.

## Abbreviations

BAC: Bacterial Artificial Chromosome; CE-LIF: Capillary Electrophoresis with Laser-Induced Fluorescence; CN: Copy Number; CNV: Copy Number Variationl; DEF: Defensin; MLPA: Multiplex Ligation Dependent Probe Amplification; PRT: Paralog Ratio Test; SNP: Single Nucleotide Polymorphism.

## Competing interests

The authors declare that they have no competing interests.

## Authors’ contributions

ST and KH designed the experiments, MG and ST carried out the data analyses, ST, KH and MP wrote the manuscript and MP coordinated the study. All authors read and approved the final manuscript.

## Supplementary Material

Additional file 1 **Table S1.** Genomic DNAs used for the length polymorphism tests and DEF cluster b CN. **Table S2.** Analyzed indels, used primers, amplicon lengths and chromosomal locations. **Table S3.** CE-LIF results for indels 3 to 7. **Table S4.** Summary of informative and non-informative samples for the proof of CNV by indels 3 to 7. **Table S5.** Allele status and chromosomal localization of clones harboring indels 1 and 2. **Figure S6.** Reduction of the number of amplified paralogs by a discriminating reverse primer (indel 1). **Figure S7.** Reduction of the number of amplified paralogs by a discriminating forward primer (indel 2). **Table S8.** Cloning and Sanger sequencing of selected PCR products from indels 1 and 2. **Table S9.** CE-LIF results for indels 1 and 2. Click here for file
