# Supplementary material for: Narrowing down the distal border of the copy number variable beta-defensin gene cluster on human 8p23
Source: BMC Res Notes. 2014 Feb 19;7:93. doi: 10.1186/1756-0500-7-93 (PMC3942070; doi:10.1186/1756-0500-7-93)
Supplement: Additional file 1 — Table S1. Genomic DNAs used for the length polymorphism tests and DEF cluster b CN. Table S2. Analyzed indels, used primers, amplicon lengths and chromosomal locations. Table S3. CE-LIF results for indels 3 to 7. Table S4. Summary of informative and non-informative samples for the proof of CNV by indels 3 to 7. Table S5. Allele status and chromosomal localization of clones harboring indels 1 and 2. Figure S6. Reduction of the number of amplified paralogs by a discriminating reverse primer (indel 1). Figure S7. Reduction of the number of amplified paralogs by a discriminating forward primer (indel 2). Table S8. Cloning and Sanger sequencing of selected PCR products from indels 1 and 2. Table S9. CE-LIF results for indels 1 and 2. [file 1756-0500-7-93-S1.pdf]

	origin	ID	diploid CN *)
1	CARLA	1105758	2
2	CARLA	1123584	2
3	CARLA	5126875	2
4	Coriell	NA12716	2
5	FLI-Met	4779	2
6	CARLA	1102560	3
7	CARLA	1102576	3
8	CARLA	1105126	3
9	CARLA	1112103	3
10	CARLA	1101938	4
11	CARLA	1103200	4
12	CARLA	1103819	4
13	CARLA	3208698	5
14	CARLA	3216315	5
15	CARLA	3216321	5
16	CARLA	5229799	6
17	CARLA	6117387	6
18	Coriell	NA12760	6
19	FLI-Met	2813	6
20	CARLA	2121707	7
21	CARLA	4107150	7
22	CARLA	1128707	8
23	CARLA	5217603	8
24	CARLA	5225985	8
25	CARLA	6132079	8

* determined by MLPA with P139 MLPA kit (MRC Holland)
CARLA= A prospective cohort study of the general elderly population,
Greiser et al. 2005 and 2009 PMID 16283930, 19199053
FLI-Met = DNA methylation study at FLI Jena,
Stepanow et al. 2011 PMID 21637341

indel	localization	indel sequence on (+)-strand	diff (bp)	amplicon's lengths (bp)		amplification primers	amplified regions and lengths with respect to hg19 = GRCh 37	paralog's localization / comment
1	DEFB109p (intron 1)	CGTT + C (putative novel)	4+1	358	363	5'-AATTCACTTGTCTGAAGTTCC-3'<br>5'-(6-FAM)-AAGTAAGGATACAACAGAACA-3'	chr8:7170725-7171082 358bp<br>chr8:7854329+7854686 358bp<br>chr12:8358585+8358947 363 bp	DEF cluster b1<br>DEF cluster b2<br>chr12, no CNV
2	273 bp upstream of start codon ATG of DEFB108p	TTGT (putative novel)	4	289	293	5'-ATACAGTAGTGGATGAGGCAAATATACA-3'<br>5'-(6-FAM)-CCTTGTTAAATGGGGACATAAGC-3'	chr8:7230128+7230416 289bp<br>chr8:7796542-7796830 289bp<br>chr11:71543706+71543998 293bp<br>chr4:9400392+9400684 293bp	DEF cluster b<br>DEF cluster b<br>chr11, no CNV<br>chr4, no CNV
							chr8:11957294-11957586 293 bp<br>chr8:12203537-12203829 293 bp	should be discriminated by primer design
3	intergenic DEF cluster b	CTGA (rs35176041)	4	270	274	5'-AAGCTGCAATGGATTCTATG-3'<br>5'-(6-FAM)-TTTTCTATTTGGCTTCCTTG-3'	chr8:7251888+7252157 270bp<br>chr8:7774792-7775061 270bp	DEF cluster b1<br>DEF cluster b2
4	SPAG11 (intron 2)	ACA (rs201835163)	3	255	258	5'-CAAGAGATGGTTGAAAGAAAG-3'<br>5'-(6-FAM)-CTGTCTTGACTAAAGGTGGAC-3'	chr8:7308949+7309203 255bp<br>chr8:7717384-7717641 258bp	DEF cluster b1<br>DEF cluster b2
5	DEFB106 (intron1)	CCCT (rs3215416)	4	392	396	5'-TTCACAAGCTGTCATAGTGC-3'<br>5'-(6-FAM)-CCTTCCAAGCAAGTTTCTATC-3'	chr8:7340366+7340761 396bp<br>chr8:7685840-7686235 396bp	DEF cluster b1<br>DEF cluster b2
6	intergenic between DEFB105 and DEFB107	AGAA (rs201857400)	4	310	314	5'-GAACACTGCACCAATTACAG-3'<br>5'-(6-FAM)-TTGTAAGTTGCTGTTGTGTTG-3'	chr8:7350141+7350454 314bp<br>chr8:7676148-7676461 314bp	DEF cluster b1<br>DEF cluster b2
7	intergenic, 10 kb proximal to DEFB107	AAAGG (rs200731641)	5	321	326	5'-ATTTTGGAAATATCTTTTAGTACTTAG-3'<br>5'-(6-FAM)-AGGATACCTACTTATTGGCTTG-3'	chr8:7376496+7376816<br>chr8:7659241-7659566	DEF cluster b1<br>DEF cluster b2

	indel	ID	Size 1	Size 2	Height 1	Height 2	Area 1	Area 2	area 1/2 ratio	informative	CN (MLPA)	Size 1	Size 2	c2813bi
1	indel_03	1102576	268	272	4,083	7,931	22,256	43,189	0.52	informative	3	1	2	1+2
2	indel_03	1105126	268	272	9,996	4,805	55,155	26,662	2.07	informative	3	2	1	2+1
3	indel_03	1112103	268	272	11,943	5,767	66,152	32,149	2.06	informative	3	2	1	2+1
4	indel_03	1102560	268	272	8,754	4,479	48,571	24,209	2.01	informative	3	2	1	2+1
5	indel_03	2813	268	272	14,523	7,281	81,043	40,602	2.00	informative	6	4	2	4+2
6	indel_03	5229799	268	271	4,345	1,847	49,900	23,528	2.12	informative	6	4	2	4+2
7	indel_03	NA12716	268	272	8,369	8,188	49,010	49,048	1.00	bi (1:1)	2	1	1	1+1
8	indel_03	1105758	268	272	8,349	8,375	46,496	46,722	1.00	bi (1:1)	2	1	1	1+1
9	indel_03	1101938	268	272	6,003	6,056	33,986	33,297	1.02	bi (1:1)	4	2	2	2+2
10	indel_03	1103200	268	272	7,035	6,873	42,311	41,572	1.02	bi (1:1)	4	2	2	2+2
11	indel_03	5126875	268	272	4,113	3,069	25,545	19,114	1.34	ambiguous	2			
12	indel_03	1103819	268		10,342		58,162			mono	4	4	0	4+0
13	indel_03	1128707	268		15,755		91,487			mono	8	8	0	8+0
14	indel_03	2121707	268		22,181		133,697			mono	7	7	0	7+0
15	indel_03	3208698	268		10,875		67,503			mono	5	5	0	5+0
16	indel_03	3216315	268		13,555		80,678			mono	5	5	0	5+0
17	indel_03	3216321	268		12,720		76,990			mono	5	5	0	5+0
18	indel_03	4107150	268		9,197		56,043			mono	7	7	0	7+0
19	indel_03	5217603	268		14,715		88,504			mono	8	8	0	8+0
20	indel_03	5225985	268		15,033		95,710			mono	8	8	0	8+0
21	indel_03	6117387	268		11,627		80,031			mono	6	6	0	6+0
22	indel_03	6132079	268		13,075		77,032			mono	8	8	0	8+0
23	indel_03	NA12760	268		14,186		87,036			mono	6	6	0	6+0
24	indel_03	4779	268		11,787		64,976			mono	2	2	0	2+0
25	indel_03	1123584		272		16,099		90,179		mono	2	0	2	0+2
1	indel_04	1112103	255	258	5,801	10,411	40,039	72,703	0.55	informative	3	1	2	1+2
2	indel_04	1102560	255	258	5,993	11,077	35,291	65,678	0.54	informative	3	1	2	1+2
3	indel_04	3216321	255	258	11,187	15,644	74,575	103,833	0.72	informative	5	2	3	2+3
4	indel_04	5229799	255	258	18,254	31,431	109,595	201,568	0.54	informative	6	2	4	2+4
5	indel_04	6117387	255	258	10,311	18,429	60,214	111,743	0.54	informative	6	2	4	2+4
6	indel_04	1105126	255	258	16,531	9,908	100,045	60,050	1.67	ambiguous	3	2	1	2+1
7	indel_04	1123584	255	258	18,071	16,684	106,524	99,994	1.07	bi (1:1)	2	1	1	1+1
8	indel_04	1105758	255	258	7,434	6,858	43,592	41,074	1.06	bi (1:1)	2	1	1	1+1
9	indel_04	5126875	255	258	12,466	15,309	72,882	88,645	0.82	bi (1:1)	2	1	1	1+1

10	indel_04	NA12716	255	258	14,847	14,242	87,654	81,304	1.08	bi (1:1)	2	1	1	1+1
11	indel_04	1101938		258		8,992		53,994		mono	4	0	4	0+4
12	indel_04	1102576		258		15,782		96,570		mono	3	0	3	0+3
13	indel_04	1103200		258		15,722		91,549		mono	4	0	4	0+4
14	indel_04	1103819		258		14,910		88,248		mono	4	0	4	0+4
15	indel_04	1128707		258		32,695		224,749		mono	8	0	8	0+8
16	indel_04	2121707		258		31,515		204,031		mono	7	0	7	0+7
17	indel_04	3208698		258		23,866		140,230		mono	5	0	5	0+5
18	indel_04	3216315		258		29,124		174,763		mono	5	0	5	0+5
19	indel_04	4107150		258		21,597		132,060		mono	7	0	7	0+7
20	indel_04	5217603		258		21,420		131,336		mono	8	0	8	0+8
21	indel_04	5225985		259		23,591		154,270		mono	8	0	8	0+8
22	indel_04	6132079		258		12,575		75,873		mono	8	0	8	0+8
23	indel_04	NA12760		258		30,999		190,978		mono	6	0	6	0+6
24	indel_04	2813		258		32,098		208,604		mono	6	0	6	0+6
25	indel_04	4779		258		29,675		182,148		mono	2	0	2	0+2
1	indel_05	1102576	390	394	2,016	3,903	13,885	27,596	0.50	informative	3	1	2	1+2
2	indel_05	1128707	390	394	2,528	5,769	18,467	39,799	0.46	informative	8	2	6	2+6
3	indel_05	3208698	390	394	3,379	4,916	24,120	35,693	0.68	informative	5	2	3	2+3
4	indel_05	1105126	390	394	4,326	2,235	30,212	15,737	1.92	informative	3	2	1	2+1
5	indel_05	1102560	391	394	5,301	2,581	37,753	18,470	2.04	informative	3	2	1	2+1
6	indel_05	5225985	390	394	4,436	5,602	32,630	42,427	0.77	informative	8	3	5	3+5
7	indel_05	4107150	390	394	2,090	3,491	16,026	26,832	0.60	informative	7	3	4	3+4
8	indel_05	NA12760	390	394	3,228	6,364	23,071	44,938	0.51	informative	6	2	4	2+4
9	indel_05	6117387	390	394	4,803	3,730	33,821	27,132	1.25	bi (1:1)	6	3	3	3+3
10	indel_05	1105758	390	394	5,638	5,621	39,523	38,604	1.02	bi (1:1)	2	1	1	1+1
11	indel_05	1123584	390	394	4,637	4,756	33,401	33,340	1.00	bi (1:1)	2	1	1	1+1
12	indel_05	1101938	390	394	2,996	3,012	21,214	21,440	0.99	bi (1:1)	4	2	2	2+2
13	indel_05	2121707	390	394	3,561	3,251	25,571	23,833	1.07	bi (1:1)	7	4	3	4+3
14	indel_05	2813	391	394	3,649	3,882	26,815	27,611	0.97	bi (1:1)	6	3	3	3+3
15	indel_05	4779	390	394	5,275	5,390	38,561	39,716	0.97	bi (1:1)	2	1	1	1+1
16	indel_05	5229799	390	394	3,073	4,083	27,112	32,400	0.84	bi (1:1)	6	3	3	3+3
17	indel_05	1112103	390		11,343		79,842			mono	3	3	0	3+0
18	indel_05	5126875	390		6,503		46,279			mono	2	2	0	2+0
19	indel_05	1103200		394		4,850		35,146		mono	4	0	4	0+4

20	indel_05	1103819		394		5,667		40,899		mono	4	0	4	0+4
21	indel_05	3216315		394		7,339		53,243		mono	5	0	5	0+5
22	indel_05	3216321		394		6,659		46,127		mono	5	0	5	0+5
23	indel_05	5217603		394		6,855		47,158		mono	8	0	8	0+8
24	indel_05	6132079		394		8,216		55,872		mono	8	0	8	0+8
25	indel_05	NA12716		394		5,848		42,672		mono	2	0	2	0+2
1	indel_06	1102560	311	315	7,003	13,577	42,081	83,676	0.50	informative	3	1	2	1+2
2	indel_06	1105126	311	315	5,981	11,627	38,237	72,980	0.52	informative	3	1	2	1+2
3	indel_06	1112103	311	315	10,816	5,206	68,368	32,993	2.07	informative	3	2	1	2+1
4	indel_06	6117387	311	315	7,150	12,066	44,002	75,744	0.58	informative	6	2	4	2+4
5	indel_06	2813	311	315	5,621	10,196	34,976	63,673	0.55	informative	6	2	4	2+4
6	indel_06	4107150	311	315	5,659	13,296	34,963	81,932	0.43	informative	7	2	5	2+5
7	indel_06	1101938	311	315	7,648	6,095	47,382	38,239	1.24	bi (1:1)	4	2	2	2+2
8	indel_06	1105758	311	315	7,282	6,939	44,169	43,631	1.01	bi (1:1)	2	1	1	1+1
9	indel_06	1123584	311	315	8,378	8,184	52,735	51,051	1.03	bi (1:1)	2	1	1	1+1
10	indel_06	4779	311	315	6,585	6,103	41,523	38,446	1.08	bi (1:1)	2	1	1	1+1
11	indel_06	5126875	311		13,214		83,292			mono	2	2	0	2+0
12	indel_06	1102576		315		9,985		63,431		mono	3	0	3	0+3
13	indel_06	1103200		315		12,990		81,564		mono	4	0	4	0+4
14	indel_06	1103819		315		14,706		90,018		mono	4	0	4	0+4
15	indel_06	1128707		315		13,331		84,709		mono	8	0	8	0+8
16	indel_06	2121707		315		18,051		111,913		mono	7	0	7	0+7
17	indel_06	3208698		315		15,111		94,998		mono	5	0	5	0+5
18	indel_06	3216315		315		19,523		123,196		mono	5	0	5	0+5
19	indel_06	3216321		315		12,959		82,692		mono	5	0	5	0+5
20	indel_06	5217603		315		17,224		107,317		mono	8	0	8	0+8
21	indel_06	5225985		315		10,374		66,793		mono	8	0	8	0+8
22	indel_06	5229799		315		13,615		91,653		mono	6	0	6	0+6
23	indel_06	6132079		315		20,491		126,171		mono	8	0	8	0+8
24	indel_06	NA12716		315		14,444		92,332		mono	2	0	2	0+2
25	indel_06	NA12760		315		14,573		92,685		mono	6	0	6	0+6
1	indel_07	3216315	319	324	1256	4301	8,201	28,378	0.288991472	informative	5	1	4	1+4
2	indel_07	4107150	319	324	96	584	611	3,738	0.163456394	informative	7	1	6	1+6
3	indel_07	5229799	319	324	3912	7545	25,408	49,517	0.513116707	informative	6	2	4	2+4
4	indel_07	NA12760	319	324	2468	5090	16,186	34,430	0.470113273	informative	6	2	4	2+4

5	indel_07	5225985	319	324	1876	4582	12,521	30,360	0.412417655	informative	8	2	6	2+6
6	indel_07	2121707	319	324	4615	5955	30,061	39,501	0.761018708	informative	7	3	4	3+4
7	indel_07	3216321	319	324	5453	3713	35,891	24,290	1.477603952	informative	5	3	2	3+2
8	indel_07	6132079	319	324	7208	4666	48,581	31,684	1.533297563	informative	8	5	3	5+3
9	indel_07	5217603	319	324	8663	3134	57,051	20,743	2.75037362	informative	8	6	2	6+2
10	indel_07	6117387	319	324	4756	4783	31,119	31,898	0.975578406	bi (1:1)	6	3	3	3+3
11	indel_07	2813	319	325	2181	2166	14,394	14,291	1.007207333	bi (1:1)	6	3	3	3+3
12	indel_07	1128707	319	324	6772	6958	44,027	46,094	0.955156853	bi (1:1)	8	4	4	4+4
13	indel_07	1101938	319	325	3933	4923	27,152	34,466	0.787790866	bi (1:1)	4	1	1	2+2
14	indel_07	1112103		325	12070			81,644		mono	3			
15	indel_07	1105126		324	6952			47,114		mono	3			
16	indel_07	1102576		324	3607			23,723		mono	3			
17	indel_07	3208698		324	3186			21,210		mono	5			
18	indel_07	1103819		324	9521			71,812		mono	4			
19	indel_07	1103200		324	12338			81,160		mono	4			
20	indel_07	5126875		325	12658			84,537		mono	2			
21	indel_07	1123584		324	3955			26,284		mono	2			
22	indel_07	1105758		325	10738			71,758		mono	2			
23	indel_07	1102560		324	14082			92,938		mono	3			
24	indel_07	4779		324	7019			46,803		mono	2			
25	indel_07	NA12716		324	4802			32,909		mono	2			

Indel	non informative tests			informative tests	
	one peak	two peaks 1:1	ambiguous	two peaks unequal 1:1	f of all
3	14	4	1	6	0.24
4	15	4	1	5	0.20
5	9	8	0	8	0.32
6	15	4	0	6	0.24
7	12	4	0	9	0.36
				34	
unique tests with two peaks unequal 1:1 peak ratios				17	0.68
tests, total				125	

indel	NCBI entry	BAC library*)	variant	map NCBI nt entry	map UCSC GRCh37 hg19	Blast vs hg19 (highest score)	Taudien 2004
indel 1	AC092111.9	RPCI-11	long	chr12	chr12	chr12:8.25Mb	na
	AC092745.7	RPCI-11	long	chr12	chr12	chr12:8.25Mb	na
	AC105214.6	RPCI-11	long	chr8	chr8:12.3Mb	chr8:12.3Mb or cluster b2	not to assign
	AC130360.4	RPCI-11	short	chr8	chr8 DEFcluster b1	chr8 DEFcluster b1	chr8 DEFcluster b1
	AF228730.8	SCb	short	chr8	chr8 DEFcluster b1	chr8 DEFcluster b1	chr8 DEFcluster b1
	AC105233.12	RPCI-11	short	chr8	chr8 DEFcluster b2	chr8 DEFcluster b2	chr8 DEFcluster b2
	AC131269.3	RPCI-11	short	chr8	na	chr8 DEFcluster b1 or b2	chr8 DEFcluster b2
	AC130339.12	RPCI-13	short	chr8	na	chr8 DEFcluster b1	chr8 DEFcluster b1
	AC130365.5	RPCI-11	short	chr8	chr8 DEFcluster b2	chr8 DEFcluster b2	chr8 DEFcluster b2
	AF252830.5	SCb	long	chr8	na	chr8 DEFcluster b2	chr8 DEFcluster b3
indel 2	AC130339.12	RPCI-13	short	chr8	na	chr8 DEFcluster b1	chr8 DEFcluster b1
	AC130360.4	RPCI-11	short	chr8	chr8 DEFcluster b1	chr8 DEFcluster b1	chr8 DEFcluster b1
	AC130365.5	RPCI-11	short	chr8	chr8 DEFcluster b2	chr8 DEFcluster b2	chr8 DEFcluster b2
	AC130367.12	RPCI-11	short	chr8	na	chr8 DEFcluster b1	chr8 DEFcluster b1
	AC131269.3	RPCI-11	short	chr8	na	chr8 DEFcluster b1 or b2	chr8 DEFcluster b2
	AC134395.7	RPCI-11	short	chr8	na	chr8 DEFcluster b1	not to assign
	AC134683.9	RPCI-11	short	chr8	na	chr8 DEFcluster b1	not to assign
	AC243982.3	CHORI-17	short	chr8	na	chr8 DEFcluster b1	na
	AF252830.5	SCb	short	chr8	na	chr8 DEFcluster b2	chr8 DEFcluster b3
	AF252831.2	SCb	short	chr8	na	chr8 DEFcluster b1	chr8 DEFcluster b1
	AF285443.4	SCb	short	chr8	na	chr8 DEFcluster b1 or b2	chr8 DEFcluster b3
	AC116655.7	CTD	long	chr4	chr4:9Mb	chr4:9Mb	na
	AC117179.4	CTD	long	chr4	na	chr4:9Mb	na
	AC130366.6	RPCI-11	long	chr8	ch8:12.0Mb	ch8:12.0Mb	na
	AC244249.3	CHORI-17	long	chr8	na	ch8:12.0Mb	na
	AC130379.8	RPCI-13	short	chr17	na	chr8:12.3Mb	na
	AC087203.12	RPCI-11	long	chr8	chr8:12.3Mb	chr8:12.3Mb	na
	AC105214.6	RPCI-11	long	chr8	chr8:12.3Mb	chr8:12.3Mb	not to assign
	AC130337.6	RPCI-13	long	chr8	na	chr8:12.3Mb	na
	AC243920.3	CHORI-17	long	chr8	na	chr8:12.3Mb	na
	AC092111.9	RPCI-11	short	chr12	chr12:8.25Mb	chr12:8.25Mb	na
	AP002495.3	CTD	long	chr11q	chr11:71.2Mb	chr11:71.2Mb	na

*) library	provider	description
RPCI-11	P.deJong, CHORI	1 male donor
RPCI-13	P.deJong, CHORI	1 female donor
CHORI-17	P.deJong, CHORI	Haploid cell line from hydatidiform mole
CTD	CalTech	1 male donor (sperm)
SCb	Research Genetics	No information available

INDEL 1

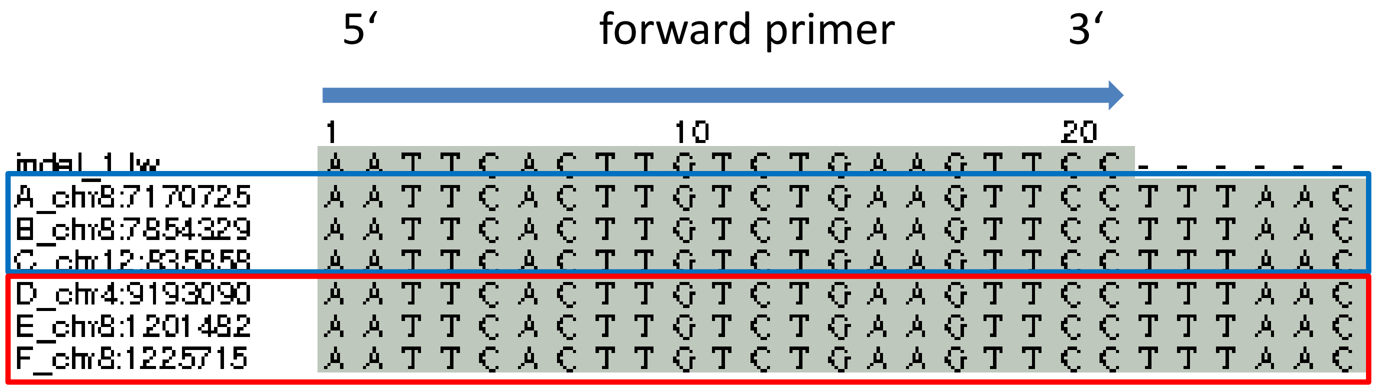

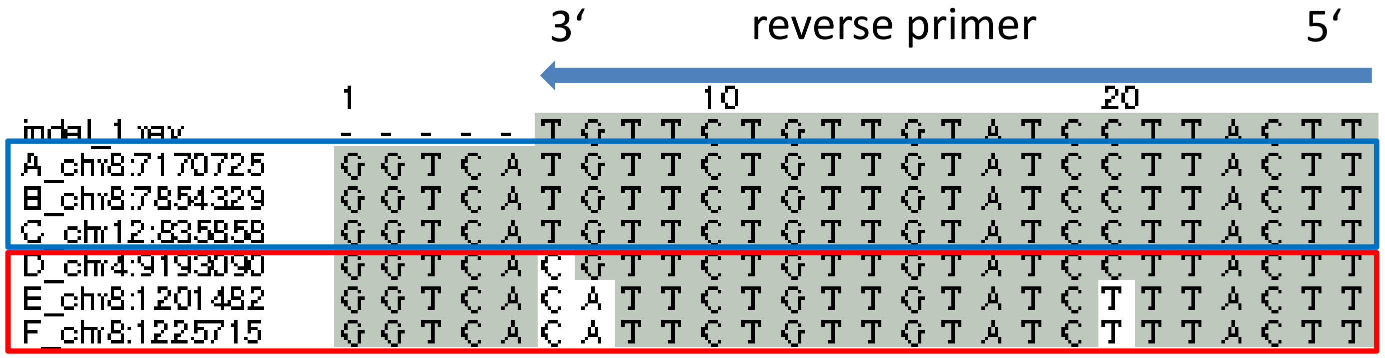

red: paralogs D_F – not amplified due to mismatches,
at 3‘ end of the reverse primer

blue: DEF cluster copies A+B, co-amplified with paralogs C

INDEL 2

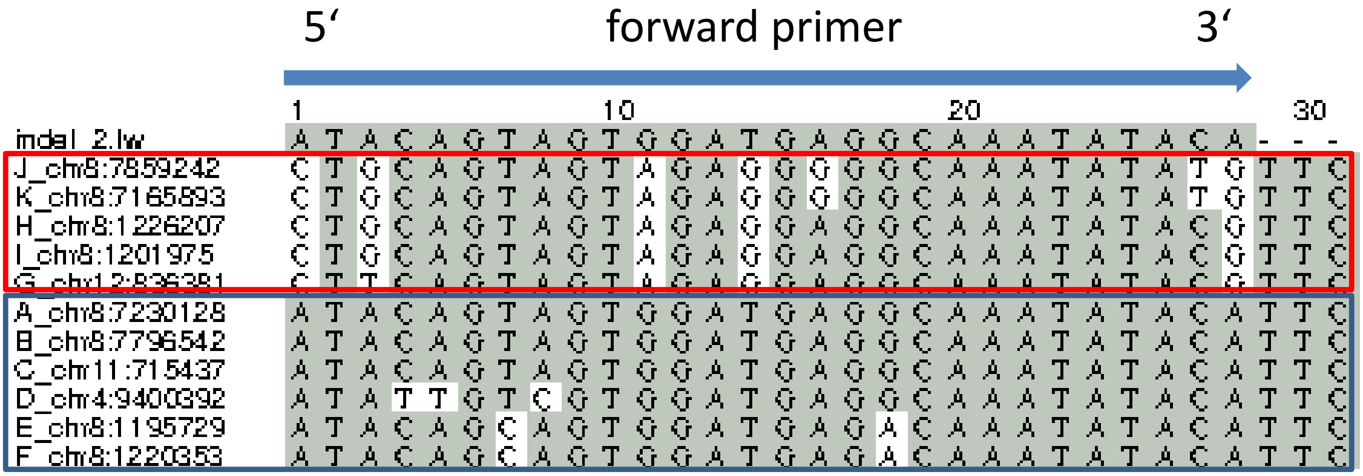

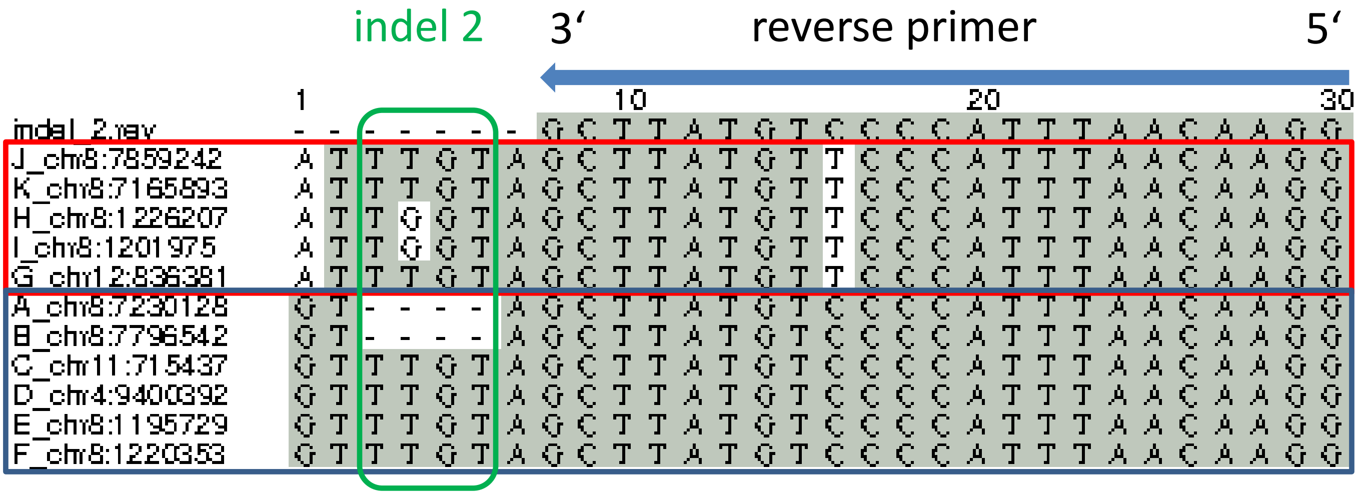

red: paralogs G-K – not amplified due to numerous mismatches, particularly at 3‘ end of the forward primer

blue: DEF cluster copies A+B, co-amplified with paralogs C-F

No. of clones identified by TOPO-TA-cloning and Sanger sequencing with non-labeled primers

indel	paralogs	hg19 positions (amplicon)	sample ID			total	variant	comment
			1105126	3208698	2121707			
1	A and B (DEF cluster)	chr8:7170725-7171082<br>chr8:7854329+7854686	20	13	22	55	short	expected to amplify
	C	chr12:8358585+8358947	8	10	6	24	long	
	D	chr4: 9193090-9193452	0	0	0	0	long	not expected to amplify due to 3' primer mismatches
	E	chr8:12014823-12015185	0	0	0	0	long	
	F	chr8:12257155-12257517	0	0	0	0	long	
	chimera		3	1	2	6	both	derived from paralogs A/B and C, no hints for paralogs D,E,F
			31	24	30	85		

indel	paralogs	hg19 positions (amplicon)	sample ID			total	variant	comment
			1101938	3216315				
2	A and B (DEF cluster)	chr8:7230128-7230416<br>chr8:7796542-7796830	2	7		9	short	expected to amplify
	C	chr11:71543706-71543998	3	1		4	long	
	D	chr4:9400392-9400684	15	19		34	long	
	E	chr8:11957294-11957586	23	7		30	long	
	F	chr8:12203537-12203829					long	
	G	chr12:8363813-8364099	0	0		0	long	not expected to amplify due to 3' primer mismatches
	H	chr8:12262077-12262362	0	0		0	long	
	I	chr8:12019754-12020039	0	0		0	long	
	J	chr8:7859242-7859522	0	0		0	long	
	K	chr8:7165893-7166173	0	0		0	long	
	chimera		0	3		3	long	derived from paralogs C and D, no hints for paralogs A,B and E-K
			43	37		80		

	indel	ID	Size 1	Size 2	Height 1	Height 2	Area 1	Area 2	area 1/2 ratio	average area 1/2 ratio per CN class	STDEV per CN class	CN (MLPA)	expected area ratio with respect to the paralogs (A+B)/C
1	indel_01	1105758	357	362	26,340	24,647	192,769	179,922	1.07	0.83094	0.26943	2	1.00
2	indel_01	1123584	357	362	15,799	14,731	116,422	114,860	1.01				
3	indel_01	5126875	358	363	22,164	28,930	166,631	258,112	0.65				
	indel_01		358	363	21,034	27,362	153,695	230,288	0.67				
4	indel_01	NA12716	358	363	13,907	29,297	114,257	242,380	0.47				
5	indel_01	4779	358	363	24,454	21,628	178,034	159,491	1.12				
6	indel_01	1102560	358	363	12,380	11,940	95,114	89,297	1.07	1.03442	0.067	3	1.50
7	indel_01	1102576	358	363	12,913	12,983	95,622	97,275	0.98				
8	indel_01	1105126	358	363	15,033	15,332	125,415	128,599	0.98				
9	indel_01	1112103	358	363	26,477	23,581	206,805	185,595	1.11				
10	indel_01	1101938	358	363	11,916	11,003	93,859	85,586	1.10	1.06191	0.08603	4	2.00
11	indel_01	1103200	358	363	5,642	4,784	42,678	37,932	1.13				
12	indel_01	1103819	358	363	16,771	17,313	135,196	140,254	0.96				
13	indel_01	3208698	358	363	22,268	22,017	162,764	163,613	0.99	1.11192	0.10	5	2.50
14	indel_01	3216315	358	363	24,629	20,948	179,896	154,528	1.16				
15	indel_01	3216321	358	363	19,885	16,851	146,337	124,354	1.18				
16	indel_01	5229799	358	363	1,297	1,317	9,275	9,508	0.98	1.02348	0.0731	6	3.00
	indel_01		358	363	1,641	1,584	15,505	15,333	1.01				
17	indel_01	6117387	358	363	28,279	29,141	201,073	207,373	0.97				
18	indel_01	NA12760	358	363	24,662	24,005	184,402	182,295	1.01				
19	indel_01	2813	358	363	11,909	10,081	86,661	75,391	1.15				
20	indel_01	2121707	358	363	1,742	1,745	12,207	12,184	1.00	0.9918	0.03184	7	3.50
	indel_01		358	363	1,507	1,454	10,870	10,496	1.04				
21	indel_01	4107150	358	363	20,158	20,516	152,222	153,503	0.99				
	indel_01		358	363	1,102	1,181	8,136	8,584	0.95				
	indel_01		358	363	2,416	2,521	17,616	17,939	0.98				
22	indel_01	1128707	358	363	29,595	27,182	210,935	198,689	1.06	1.06009	0.04921	8	4.00
23	indel_01	5217603	358	363	24,819	21,875	179,543	157,962	1.14				
24	indel_01	5225985	359	363	1,765	1,738	12,277	12,180	1.01				
	indel_01		358	363	1,520	1,486	10,696	10,410	1.03				
25	indel_01	6132079	358	363	26,264	24,627	190,285	178,381	1.07				

	indel	ID	Size 1	Size 2	Height 1	Height 2	Area 1	Area 2	area 1/2 ratio	average area 1/2 ratio per CN class	STDEV per CN class	CN (MLPA)	expected area ratio with respect to the paralogs (A+B)/(C+D)
1	indel_02	NA12716	289	293	265	692	1,650	4,874	0.34	0.46	0.07869	2	0.50
2	indel_02	4779	289	293	1,143	2,316	6,994	15,549	0.45				
3	indel_02	1123584	289	293	1,437	2,824	9,343	19,309	0.48				
4	indel_02	1105758	289	293	1,745	3,249	11,246	22,599	0.50				
5	indel_02	5126875	289	293	5,273	8,558	32,254	58,703	0.55				
6	indel_02	1102576	289	293	13,187	18,425	82,045	127,630	0.64	0.71	0.07712	3	0.75
7	indel_02	1105126	289	293	5,786	8,106	37,520	57,101	0.66				
8	indel_02	1112103	289	293	3,906	4,724	24,631	33,689	0.73				
9	indel_02	1102560	289	293	32,079	32,207	289,634	357,389	0.81				
10	indel_02	1101938	289	293	22,835	26,795	144,103	184,558	0.78	0.86	0.0669	4	1.00
11	indel_02	1103200	289	293	32,385	32,413	255,832	286,257	0.89				
12	indel_02	1103819	289	293	31,369	31,506	238,568	265,241	0.90				
13	indel_02	3208698	289	293	2,676	2,448	16,051	17,171	0.93	1.12	0.16049	5	1.25
14	indel_02	3216315	289	293	1,877	1,287	11,815	9,804	1.21				
15	indel_02	3216321	289	293	2,561	1,868	16,615	13,621	1.22				
16	indel_02	2813	289	293	918	848	6,284	7,203	0.87	1.17	0.22589	6	1.50
17	indel_02	NA12760	289	293	1,020	732	6,698	5,835	1.15				
18	indel_02	5229799	289	293	1,596	1,109	9,685	7,551	1.28				
19	indel_02	6117387	289	293	1,310	892	7,920	5,670	1.40				
20	indel_02	4107150	289	293	2,596	1,683	16,755	11,651	1.44	1.46	0.03478	7	1.75
21	indel_02	2121707	289	293	4,901	3,022	30,065	20,215	1.49				
22	indel_02	5225985	289	293	5,636	3,351	34,699	22,172	1.56	1.80	0.2444	8	2.00
23	indel_02	1128707	289	293	6,169	3,137	38,793	23,550	1.65				
24	indel_02	6132079	289	293	3,299	1,636	20,684	11,071	1.87				
25	indel_02	5217603	289	293	3,464	1,528	21,440	10,162	2.11				
